# Biological Response of Osteoblasts to Zirconia Manufactured via FFF, DLP, and Milling

**DOI:** 10.3390/jfb16110397

**Published:** 2025-10-23

**Authors:** Christoph J. Roser, Ralf Erber, Andreas Zenthöfer, Stefan Rues, Christopher J. Lux, Dorit Nötzel, Ralf Eickhoff, Thomas Hanemann

**Affiliations:** 1 Department of Orthodontics and Dentofacial Orthopedics, Heidelberg University Hospital, Im Neuenheimer Feld 400, 69120 Heidelberg, Germany; ralf.erber@med.uni-heidelberg.de (R.E.); christopher.lux@med.uni-heidelberg.de (C.J.L.); 2 Department of Prosthodontics, Heidelberg University Hospital, Im Neuenheimer Feld 400, 69120 Heidelberg, Germany; andreas.zenthoefer@med.uni-heidelberg.de (A.Z.);; 3 Institute for Applied Materials, Karlsruhe Institute of Technology, Herrmann-von-Helmholtz-Platz 1, 76344 Eggenstein-Leopoldshafen, Germany; dorit.noetzel@kit.edu (D.N.); ralf.eickhoff@kit.edu (R.E.); thomas.hanemann@kit.edu (T.H.)

**Keywords:** additive manufacturing, FFF, FDM, zirconia, implant, 3D printing, CAD/CAM, osteoblast

## Abstract

(1) Background: Zirconia (ZrO_2_) is increasingly used in dental implantology due to its biocompatibility and favorable mechanical and biological properties. While subtractive and stereolithographic additive manufacturing techniques are well established, the application of Fused Filament Fabrication (FFF) for zirconia-based dental implants remains largely unexplored. (2) Methods: Cylindrical ZrO_2_ specimens were fabricated using three different manufacturing techniques: milling (MIL), Digital Light Processing (DLP), and FFF. Surface topography was analyzed via white-light interferometry. Human fetal osteoblasts (hFOBs 1.19) were cultured on the specimens to evaluate cell adhesion after 4 and 24 h, proliferation for 4 days, cell surface coverage after 4 and 24 h, and osteogenic gene expression (RUNX2, ALPL, and BGLAP) after 24 h, 48 h, 7 days, and 14 days. (3) Results: The FFF samples exhibited significantly higher surface roughness than the MIL and DLP specimens. After 24 h, enhanced cell adhesion and the highest proliferation rates were observed on FFF surfaces. At 14 days, gene expression analysis revealed elevated expression of BGLAP on FFF surfaces, suggesting advanced osteogenic differentiation compared to MIL and DLP. (4) Conclusions: The inherent surface roughness of FFF-printed zirconia appears to promote osteogenic activity without additional surface treatment. These findings suggest that FFF may constitute a viable manufacturing method for the fabrication of customized zirconia components in dental implantology, warranting further investigations, particularly regarding their mechanical performance.

## 1. Introduction

Due to its numerous benefits, like biocompatibility [1], decreased biofilm formation [2], marginal concentration, the lower wear-induced cytotoxicity of its nanoparticles [3], and the reduced inflammatory reaction it provokes under induced peri-implantitis [4], zirconia (ZrO_2_) has gained attention in the field of oral surgery concerning the production of dental implants. Thus, ZrO_2_ implants now have mid-term survival and success rates comparable to those of titanium (Ti) implants [5,6,7].

In terms of manufacturing ZrO_2_, milling still represents the current standard approach. However, using milling techniques for ZrO_2_ limits its possibilities, especially with respect to the production of complex and particular filigree geometries. This is because milling is always limited by the shapes of the milling instruments used and the axes of computer numerical control (CNC) machines with restricted operating ranges. Moreover, the milling of ZrO_2_ can lead to defects like cracks during the manufacturing process, dubbed tooling stress [8,9,10,11], and is always associated with high levels of material consumption.

To overcome these limitations, the additive manufacturing (AM) of ZrO_2_ has been introduced as an alternative. Using AM facilitates the manufacture of small and complex geometries like hollow parts, mesh structures, undercuts, and micro-structured surfaces [12] and porosities tailored to individual patients [13]. The most representative methods used for the AM of dense ceramics are stereolithographic printing methods like Digital Light Processing (DLP) and material extrusion methods like Fused Filament Fabrication (FFF), also known as Fused Deposition Modeling (FDM). Although DLP for the AM production of ZrO_2_ might be promising due to the feasibility of producing fine details, it entails high investment and maintenance costs. The FFF of ZrO_2_ may overcome these limitations as it combines high material efficiency with reduced consumption costs and the ability to manufacture geometries with fine details [14,15,16].

To create dense, stiff, and strong structures, all ceramic materials must be postprocessed. In the case of DLP and FFF, the binder of the green body must be removed (a process known as debinding) before the structure is densified via sintering. However, due to the various polymers within the green bodies, the debinding of thick-walled (>1 mm) FFF parts can be a faster method than the DLP approach.

**Figure 1:**
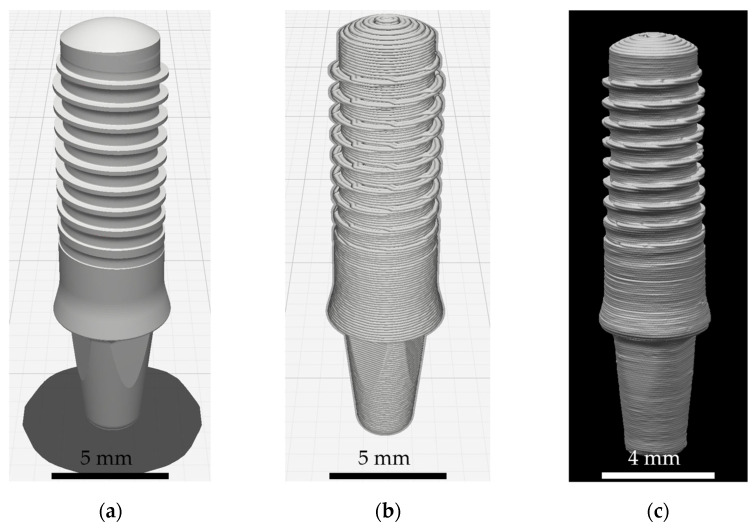
Dental implant produced via Fused Filament Fabrication: (**a**) model, (**b**) layers to be printed after slicing, and (**c**) printed and postprocessed implant.

While the use of DLP in dental applications is commonly known, using FFF to produce dental implants is only discussed as a potential method in the literature [17,18,19]. Furthermore, to the best of our knowledge, no studies have investigated FFF in this context to date. This printing method is remarkable for two reasons: (i) It does not necessitate the printing of fully filled bodies. If the mechanical properties are not affected, cavities can reduce material costs and printing time. (ii) As this printing strategy involves the use of nozzles and a coarse layer height, in comparison to DLP, FFF-printed bodies show a native macroscopic surface structure that is associated with higher surface roughness. For illustration, Figure 1a displays the design of a dental implant to be printed. In Figure 1b, the body is subdivided into layers with a printing high of 100 µm. Figure 1c shows the resulting FFF-printed and postprocessed implant with a clear visible morphology.

Several studies have investigated how surface morphology affects cell growth on various materials [20,21]. Furthermore, it has been found that cells are influenced by mechanical stresses due to 3D microenvironments [22]. Due to the distinctive surface structures of FFF-printed samples, the response of human fetal osteoblasts is very interesting.

In our previous study, we performed an investigation comparing DLP-ZrO_2_ to milled ZrO_2_ (Mil-ZrO_2_). In this study, we extend our investigation to FFF-ZrO_2_ and investigate its osteogenic potential by studying its osteoblast adhesion, coverage, and proliferation and the expression of osteogenic marker genes (RUNX2, ALPL, and BGLAP). Essentially, our ongoing work focuses on the qualitative characterization of osteoblast activity, aiming to gain insight into the processes underlying osseointegration and guide future research on AM-ZrO_2_ implants.

## 2. Materials and Methods

### 2.1. Production of Specimens and Quantification of Respective Surface Roughness

For all the groups, cylindrical specimens (with the following dimensions after sintering: diameter = 5 mm, and thickness = 2 mm) were designed digitally using CAD-Software Geomagic Design X 2022.1 (3D Systems, Rock Hill, SC, USA). Then, the specimens were produced using the corresponding manufacturing technique. For the DLP- and FFF-printed materials, polymers were used as temporary binders for shaping. During postprocessing, these binders were removed, so all the tested samples consist of 3 mol% yttria-stabilized tetragonal ZrO_2_. After all the specimens were produced, they were allocated to the three test groups according to how they were manufactured (Mil, DLP, or FFF).

#### 2.1.1. Milled Specimens

The milled specimens were manufactured from 3 mol% yttria-stabilized tetragonal ZrO_2_ polycrystal (e.max ZirCAD LT, Ivoclar Vivadent, Schaan, Liechtenstein) using a 5-axis milling device (PM7, Ivoclar Vivadent). Afterwards, sintering was performed according to the manufacturer’s recommendations (Programat S1 1600, Ivoclar Vivadent).

#### 2.1.2. AM DLP Specimen

The AM specimens were manufactured via a DLP printer (CeraFab 7500; Lithoz, Vienna, Austria) using a 3D-printable 3 mol% yttria-stabilized tetragonal ZrO_2_ polycrystal material (LithaCon 3Y 230, Lithoz) containing approximately 60 vol% of polymers. The nesting orientation, i.e., the orientation on the printing platform, was horizontal, as with the FFF specimen. DLP printing was performed using the following parameters: layer height, 25 µm; DLP energy, 190 mJ/cm^2^; and intensity, 96.9 mW/cm^2^. After the printing process, all DLP-specimens were cleaned from slurry remnants (LithaSol30; Lithoz; air pressure) and postprocessed (debinding and sintering) as previously described [23] using a furnace (HTCT 08/16; Nabertherm, Lilienthal, Germany). Debinding and presintering were performed at temperatures up to 1000 °C. In this state, individual color infiltration and staining could take place in a dental workflow. This step was, however, omitted in this investigation. Subsequent sintering until full density was reached was performed at 1450 °C (with the specimens kept at the final temperature for 2 h).

#### 2.1.3. AM FFF Specimen

**Figure 2:**
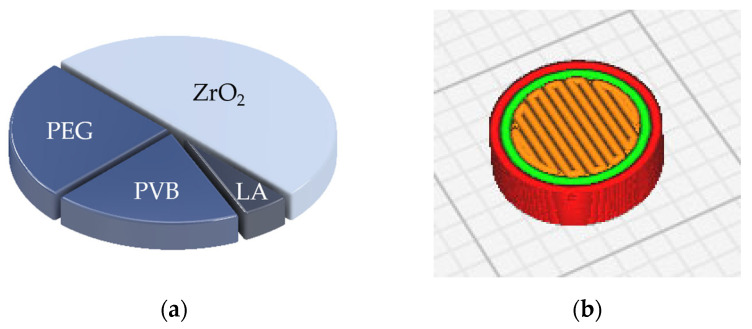
(**a**) Feedstock composition in volume percentage (50 vol.% zirconia (ZrO_2_), 15.0 vol.% PVB (polyvinyl butyral), 27.5 vol.% PEG (polyethylene glycol), and 7.5 vol.% LA (lauric acid)). (**b**) Horizontal nesting orientation in slicer software. Red lines, outer-side surface; green lines, inner walls; orange lines, top surface.

In FFF, the working material used was a ceramic-loaded thermoplastic feedstock, which was developed in-house based on Nötzel et al.’s publication [24]. The alumina ceramic was replaced with ZrO_2_ ceramic (TZ-3YS-E, Tosoh, Tokyo, Japan), and stearic acid (SA) was replaced with lauric acid (LA). Figure 2a shows the composition in volume percent.

The thermoplastic polymers used served as vehicles for shaping the ceramic particles plastically. Polyvinyl butyral (PVB, B30H, Kuraray Europe GmbH, Hattersheim am Main, Germany) accounts for the mechanical strength of the printed part but has a high melting temperature and viscosity. Polyethylene glycol (PEG, m_w_ 4000, Roth GmbH, Karlsruhe, Germany) decreases the melting point and viscosity of the mixture. Lauric acid (LA, Carl Roth GmbH, Karlsruhe, Germany) deagglomerates the primary particles of the ceramic powders and decreases the viscosity of the feedstock.

The materials were homogenized in a laboratory internal mixer (W50 EHT, Brabender, Duisburg, Germany) for 60 min at 110 °C. Filaments were extruded using a single-screw filament extruder (Noztek pro HT, Noztek, Shoreham, UK). The specimens were sliced using Ultimaker Cura (Ultimaker B.V., Utrecht, The Netherlands) and then printed using a slightly modified FFF printer (X350 pro, German Reprap GmbH, Feldkirch, Germany). The modification of the print head is described in detail elsewhere [25]. In contrast to the latest printers on the market, the direct extruders of the printers used when this investigation was conducted still needed to be modified for the purposes of this investigation. The orientation of the specimen during printing is shown in Figure 2b. The main printing conditions were a nozzle diameter of 400 µm and a layer height of 100 µm. The nozzle and printing bed temperatures were 170 and 60 °C.

The different kinds of polymers allow for two-step debinding. PEG was dissolved in water at ambient temperature and then subjected to thermal debinding. More precise information about the printing and debinding conditions can be found in [24]. Sintering was performed in a chamber oven in ambient air at a temperature of 1450 °C (RHF17/3, Carbolite, Neuhausen, Germany), with the specimen lying on an alumina substrate.

#### 2.1.4. Control Surfaces/Tissue Culture Plastic

Control experiments for evaluating cell adhesion, coverage, and differentiation were conducted on standard cell culture plastics (polystyrene). All cell culture wares—including flasks, dishes, and multi-well plates—were obtained from Greiner Bio-One GmbH, Frickenhausen, Germany, and used according to the manufacturer’s specifications.

### 2.2. Surface Morphology

In order to reveal the differences in surface morphology between the three fabrication methods, one randomly selected sample from each group was measured using a white-light interferometer (Fries Research Technology GmbH, Wetzlar, Germany) on an area of 1500 × 2000 µm^2^ according to DIN EN ISO 25178-602. The light source used was a 100 W halogen lamp (Osram GmbH, Munich, Germany), and sensor parameters of 320 Hz and a minimal intensity of 50 were used. The resolution was 5 µm^2^, and the S and L filters were 100 and 300 µm. An F operator was not used.

### 2.3. Cell Culture

Human fetal osteoblasts (hFOBs 1.19; CRL-11372, American Culture Collection, ATCC^®^, Manassas, VA, USA) were cultured in a 1:1 (vol./vol.) mixture of Dulbecco’s Modified Eagle Medium (DMEM) with 2.5 mM L-glutamine and Ham’s F-12 Medium. This culture medium was supplemented with 10% fetal bovine serum, 100 IU/mL of penicillin, 100 µg/mL of streptomycin, 2.5 mg/mL of amphotericin B, and 300 µg/mL of G418 (all reagents were obtained from Bio&Sell, Feucht, Germany). The cells were maintained at a permissive temperature of 34 °C in a humidified incubator containing 5% CO_2_.

### 2.4. Cell Adhesion and Proliferation

The adhesion of hFOB 1.19 cells onto ZrO_2_ surfaces was evaluated using the CellTiter 96^®^ assay (Promega, Mannheim, Germany). ZrO_2_ specimens were placed in 96-well plates, and 10,000 cells per wall were seeded in 200 μL of culture medium for 4 h and 24 h under conditions of 34 °C and 5% CO_2_. To specifically assess cell adhesion on the specimens, which have a slightly smaller diameter than the wells, the specimens were gently removed using Dumont forceps (#7, tip size 0.07 × 0.04 mm^2^, F.S.T., Heidelberg, Germany) without making contact with the upper surface and transferred to a second plate containing wells prefilled with 100 μL of culture medium. The CellTiter 96^®^ assay was performed according to the manufacturer’s protocol. Optical density (OD) was measured with a multi-well reader (GeniosPro, Tecan, Crailsheim, Germany) at a wavelength of 490 nm.

To determine relative cell adhesion, we calculated the results as the ratio of the ODs of the ZrO_2_ specimens to the ODs of the positive controls (hFOB 1.19 cells cultured on tissue culture plastic (TCP) surfaces), adjusted for the smaller surface area of the ZrO_2_ specimens. Cell adhesion on the ZrO_2_ specimens was expressed as a percentage relative to the TCP controls (control = 100%).

A similar protocol was employed to assess cell proliferation on the ZrO_2_ specimens. For this purpose, cell counts were recorded after 4, 24, 48, 72, and 96 h, with the 4 h value defined as 100% for the measurement series. All experiments were conducted on nine surfaces or controls in triplicate, yielding a total of 27 samples per material (and control) at each time point available for statistical analysis.

### 2.5. Cell Coverage

Initially, 10,000 hFOB cells per well were seeded on ZrO_2_ specimens placed in 96-well plates. After an incubation period of 24 h, the cells were washed three times with phosphate-buffered saline (PBS) and subsequently fixed with 2.0% glutaraldehyde in PBS at 4 °C for 16 h. After fixation, the specimens were equilibrated to room temperature and subjected to a dehydration series using increasing ethanol concentrations (70%, 80%, 90%, and 100%). Each dehydration step was followed by 3 min incubation in a graded ethanol/hexamethyldisilazane (HMDS) series: 2:1 ethanol/HMDS, 1:2 ethanol/HMDS, and finally pure HMDS. The samples were then air-dried.

To thoroughly evaluate the interactions between human fetal osteoblasts and ZrO_2_ specimens, scanning electron microscopy (SEM) was employed. SEM analysis was conducted without sputter coating utilizing a field emission scanning electron microscope (Ultra 55; Zeiss, Oberkochen, Germany) operated at 1 kV. Additionally, backscattered electron detector (BSED) images were captured at 1.5 kV. Two independent researchers performed the analyses, focusing on cellular spreading and initial contact with the ZrO_2_ material.

To quantitatively assess cell spreading, six samples of both ZrO_2_ and control surfaces were examined. SEM images at 550× magnification were analyzed using ImageJ software Version 1.54p (Rasband, W.S., ImageJ, U.S. National Institutes of Health, Bethesda, MD, USA). Three representative regions of interest (ROIs) from each of the six samples per surface were selected for analysis. The parameters measured included the number of cells, the area covered by cells, and the total area within the ROI. Cell coverage was calculated using the following formula: (area covered by cells within the ROI/the number of cells within the ROI)/total area of the ROI. Three ROIs were analyzed for each of the six surface or control samples, resulting in a total of 18 datapoints per surface for statistical evaluation. This meticulous analysis provided detailed insights into the interaction dynamics between hFOBs and the ZrO_2_ surfaces.

### 2.6. RT-PCR Analysis

For the quantitative reverse transcription polymerase chain reaction (qRT-PCR) analysis, cells were cultured on twelve ZrO_2_ specimens per surface condition or in twelve wells of 96-well plates as controls. Total RNA was extracted from the pooled cells obtained from these twelve specimens or wells by using the RNeasy Kit (Qiagen, Hilden, Germany) to ensure sufficient yield. The integrity of the isolated RNA was verified via capillary electrophoresis using the Experion System (Bio-Rad, Munich, Germany).

After RNA extraction, the total RNA was reverse-transcribed into complementary DNA (cDNA) using recombinant Moloney Murine Leukemia Virus Reverse Transcriptase (M-MuLV RT) and poly-dT (Polydeoxythymidin) primers (Thermo Fisher Scientific, Dreieich, Germany). The synthesized single-stranded cDNA was then utilized for qPCR analyses, which were performed in technical triplicates for each condition.

Quantitative PCR was conducted using SYBR Green chemistry (Bio-Rad, Munich, Germany) via an iCycler Instrument (Bio-Rad, Munich, Germany). To ensure consistent and accurate amplification efficiencies, we utilized predesigned RT^2^ qPCR Primer Assays (Qiagen, Hilden, Germany), with human GAPDH serving as the reference gene. Detailed information on the assay IDs can be found in Table 1 below.

The relative gene expression levels were quantified using the delta-delta CT (ΔΔCT) method, as described by Livak and Schmittgen [26]. This method allows for the comparison of gene expression between different samples, normalized to the reference gene and relative to a control sample. The rigorous and systematic approach to qRT-PCR employed in this study ensured the gene expression data are highly accurate and reproducible.

### 2.7. Statistical Analysis

All data are expressed as mean values accompanied by the standard deviation (mean ± SEM). To compare differences between the experimental surface groups, the non-parametric Kruskal–Wallis one-way analysis of variance on ranks was applied, as the data did not meet the assumptions required for a parametric ANOVA. When a significant overall difference was detected via the Kruskal–Wallis test, pairwise group comparisons were conducted using Dunn’s post hoc test to identify which specific groups differed from each other. Statistical analyses were carried out using SigmaPlot software version 14 (Systat Software GmbH, Erkrath, Germany). A *p*-value less than 0.05 was considered indicative of a statistically significant difference.

## 3. Results

### 3.1. Surface Roughness Quantification

**Figure 3:**
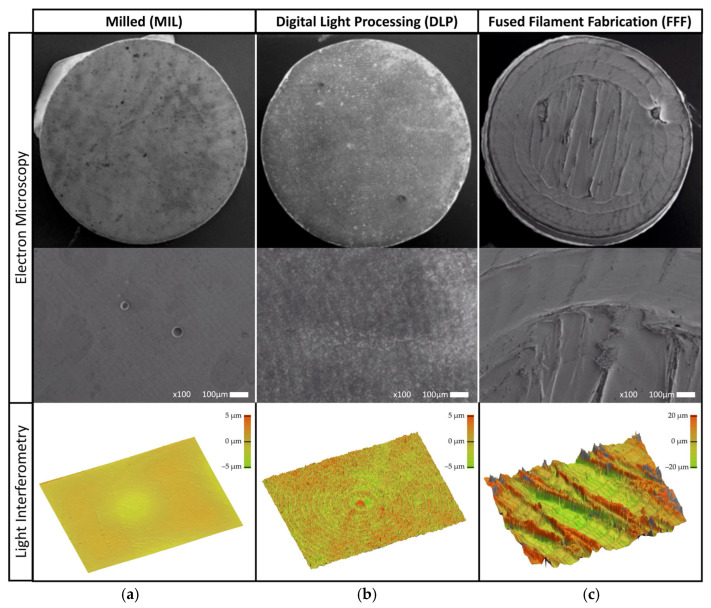
Electron microscope images and white-light interferometer images (1 × 2 mm^2^): (**a**) MIL, (**b**) DLP, and (**c**) FFF.

The surfaces resulting from the different manufacturing methods are shown in Figure 3. From top to bottom, the magnification increases from an overview of one randomly chosen sample to a higher-resolution image taken in SEM. The lower row displays false-color images of the topography, with an area of 1 × 2 mm^2^.

The MIL sample (Figure 3a) shows the circular path of the milling tool during manufacturing, while the DLP sample (Figure 3b) seems to have a very smooth surface. These features are visible because of the simultaneous lighting of the whole top area in this geometry. In contrast, the FFF sample (Figure 3c) has very pronounced surface structures arising from the path of the printing tool. Depositing material extruded through a nozzle results in characteristic textures within the printing plane [27,28]. These textures follow the tool path of the printer head that fills the layers line by line.

Table 2 shows the results for surface quantification. The MIL (Sa: 0.3 µm) and DLP (Sa: 0.2 µm) samples show comparable mean heights in terms of surface roughness. In contrast, the FFF sample exhibits a Sa of 5.5 µm, with significantly higher surface roughness.

The RMS (Root Mean Square) value for height, Sq, corresponds to the Sa in an unsurprising way. Except for the milled sample, the maximum height, Sz, is much higher than the maximum peak height, Sp. This difference could be the result of an issue relating to the measuring method. Some areas that appear very deep seem to be a misinterpretation on the part of the software.

The surface roughness of the FFF-printed parts is a product of the described surface topographies and influenced by layer height, build orientation, and the nozzle diameter [8]. The side surfaces of the FFF-printed parts (Figure 2b, red lines) have even greater surface roughness than the top surfaces (yellow plane in Figure 2b) [29,30,31]. In this study, only the top surfaces of the disks were analyzed because cell-growing tests (adhesion, proliferation, and coverage) were carried out on these planes.

### 3.2. Cell Adhesion on Zirconia Surfaces

**Figure 4:**
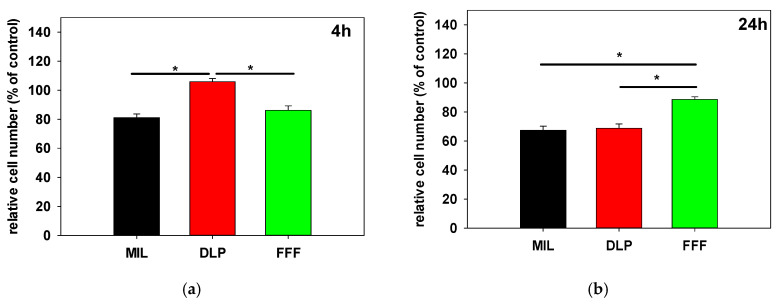
Cell adhesion of hFOB 1.19 on zirconia surfaces relative to tissue culture plastic (100%) after (**a**) 4 h and (**b**) 24 h. Adhesion was highest on the DLP surfaces after 4 h but decreased markedly by 24 h, while the FFF surfaces maintained a constant level and even exhibited slightly increased adhesion, resulting in significantly higher cell attachment on the FFF surfaces compared to the DLP and MIL surfaces after 24 h; * *p* < 0.05, Kruskal–Wallis One-Way Analysis of Variance on Ranks, Bonferroni post hoc test, n = 9.

Cell adhesion of hFOB 1.19 on the ZrO_2_ surfaces was measured relative to cell adhesion on tissue culture plastic (control) which was arbitrarily set to 100%. The data on adhesion to the ZrO_2_ surfaces are shown graphically in Figure 4. Adhesion after 4 h was significantly higher on the DLP (mean ± SD, 105.9% ± 6.5) surfaces relative to the FFF (86.2% ± 9.2) and MIL (81.2% ± 7.5) surfaces (Figure 4a). Notably, after 4 h, the adhesion of hFOB 1.19 slightly exceeded adhesion on the tissue culture plastic. However, after 24 h, the DLP sample also experienced the largest decrease (−37.1%) in adherent cells between to then 68.8% ± 5.4 (Figure 4b). Adherence also decreased on the MIL sample (−13.8% to 67.4% ± 8.4), while a slight increase was observed on the FFF surfaces (+2.5% to 88.7% ± 5.4). The loss of adherent cells on both the DLP and MIL surfaces combined with the slightly improved adherence on the FFF surfaces led to significantly better adherence of hFOB 1.19 on the FFF surfaces compared to the DLP and MIL surfaces after 24 h. The observed decreases regarding adherent cells on the DLP and MIL surfaces within the observation period might be due to secondary detachment of cells that is not compensated for by cell proliferation, while increases in adherent cells could depend on increased proliferation of cells on the FFF surface. To evaluate this aspect, we measured the proliferation of hFOB cells on the different zirconia surfaces in a further experiment over a period of 4 days.

### 3.3. Cell Proliferation on Zirconia Surfaces

**Figure 5:**
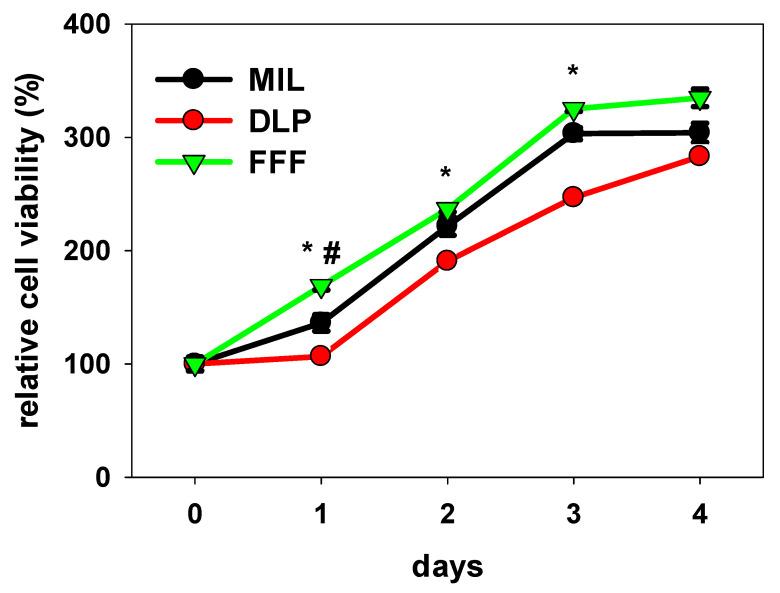
Proliferation of hFOB 1.19 cells on zirconia surfaces. The data on proliferation are presented relative to the number of cells measured on the respective surfaces at the start of the monitoring period (t0 = 4 h after the seeding of the cells), with value arbitrarily designated as 100%. Significant differences were observed for FFF-ZrO_2_ versus Mil-ZrO_2_ and DLP-ZrO_2_ on day 1 and between FFF-ZrO_2_ and SLA-ZrO_2_ on days 2 and 3. * *p* < 0.05, FFF vs. DLP, # *p* < 0.05 FFF vs. MIL, Kruskal–Wallis One-Way Analysis of Variance on Ranks, Bonferroni post hoc test, n = 9.

To elucidate the differences between the zirconia surfaces with respect to cell proliferation, cell numbers were obtained after 4 h (0) and then every 24 h for a total of 96 h. Figure 5 presents the proliferation data relative to the number of cells measured on the respective surfaces at the start of the monitoring, which was arbitrarily designated as 100%. Cells proliferated on all the surfaces. On the MIL and FFF surfaces, the cells already seemed to reach a growth plateau, presumably because the cell density in connection with the dwindling supply of nutrients and limited growth area was already too high, preventing further proliferation. Between t0 and day 3, proliferation on the MIL and FFF surfaces was superior to that on the DLP surfaces. Significant differences were observed regarding FFF versus MIL and DLP on day 1 and concerning FFF versus DLP on days 2 and 3. Interestingly, proliferation on FFF reached the minimal doubling time of 36 h documented for hFOB 1.19 [32]. Thus, the observed advantage in terms of cell adhesion after 24 h for FFF may be partly due to the support of hFOB1.19 cell proliferation by this surface.

### 3.4. Cell Coverage

**Figure 6:**
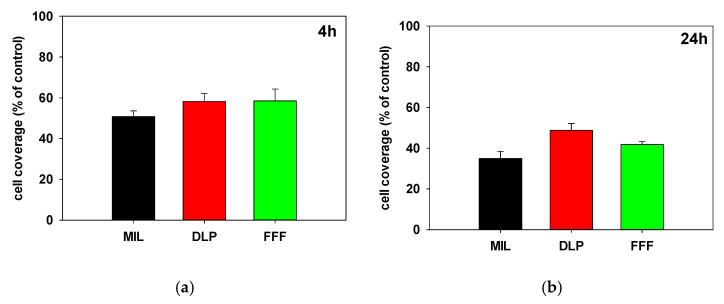
Cell coverage of hFOB 1.19 cells on zirconia surfaces after (**a**) 4 h and (**b**) 24 h. Cell coverage was evaluated using scanning electron microscopy (SEM). Cells were cultured on ZrO_2_ surfaces (MIL = milled, DMP = Digital Light Processing, and FFF = Fused Filament Fabrication) and standard cell culture plastic (as a control) for 4 h (**a**) or 24 h (**b**), respectively. For each group, six specimens were analyzed to assess cell coverage. Cell coverage was quantified as the area occupied by cells within a defined region of interest (ROI), normalized by the number of cells in the ROI and the total area of the ROI. No statistically significant differences in cell coverage were observed between the ZrO_2_ surfaces.

Surface topography characteristics influence cell-surface interactions, leading to distinct cellular morphologies, which ultimately lead to different surface areas of cell populations. To evaluate the impact of the different zirconia surfaces on cell morphologies, we measured cell coverage, which depends, given the similar cell numbers, on individual cell spreading. Cell coverage, defined using the formula (area covered by cells within the ROI/the number of cells within the ROI)/total area of the ROI, was measured using scanning electron microscopy after the cells had grown on the zirconia surfaces for 4 h and 24 h. Cell coverage on tissue culture plastic, with the value arbitrarily set to 100%, was also analyzed, and the cell coverage values determined on the zirconia surfaces are depicted relative to the cell coverage determined on tissue culture plastic (Figure 6). After 4 h, the extent of cell coverage on the zirconia surfaces was significantly worse relative to the tissue culture plastic; however, between the zirconia surfaces, no significant differences were evident. The extent of cell coverage was almost similar on the FFF (58.5%) and DLP (58.2%) surfaces, while the coverage on the MIL surfaces was slightly lower (50.8%). After 24 h, slightly greater coverage was observed for DLP (48.8%) compared to FFF (41.7%) and MIL (34.9%). Again, between the zirconia surfaces, significant differences in cell coverage could not be determined.

### 3.5. Gene Expression Analysis via Real-Time Quantitative RT-PCR

**Figure 7:**
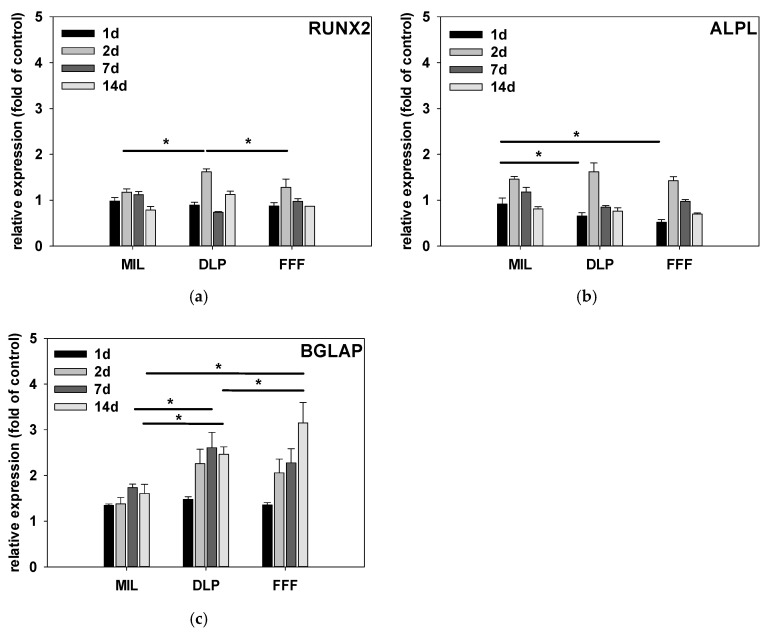
Expression of osteogenic markers. Temporal expression of osteogenic marker genes in hFOB 1.19 cells cultured on different zirconia surfaces. Cells were incubated for 1 d, 2 d, 7 d, and 14 d, and the expression of the early differentiation markers RUNX2 (**a**) and ALPL (**b**), as well as the late marker osteocalcin (BGLAP) (**c**), was quantified using qRT-PCR and normalized to the values for tissue culture plastic. RUNX2 and ALPL expression peaked after 2 d, with the highest expression observed on DLP surfaces. Osteocalcin expression peaked at later time points (7–14 d), with the highest levels observed on FFF surfaces and the lowest observed on MIL surfaces, indicating a regular osteogenic differentiation pattern and more pronounced late-stage differentiation on the FFF and DLP surfaces. * *p* < 0.05, Kruskal–Wallis One-Way Analysis of Variance on Ranks, Bonferroni post hoc test, n = 3.

Osteoblast differentiation can be monitored via the temporal expression patterns of specific marker genes. In order to determine the potential influence of zirconia surfaces on hFOB 1.19 differentiation, we cultured the cells at 39 °C, i.e., with inactive Tag for 1 day, 2 days, 7 days, and 14 days, and examined the expression of the early differentiation markers RUNX2 and ALPL as well as the late marker osteocalcin (BGLAP) using quantitative PCR. The results are shown graphically in Figure 7. The expression is shown normalized to cells that were cultured on tissue culture plastic, with the value arbitrarily set to 100%. As expected, the expression of the early differentiation markers RUNX2 (Figure 7a) and ALPL (Figure 7b) peaked after 2 days on all the zirconia surfaces. The levels of expression of both RUNX2 and ALPL were highest on the DLP surfaces. However, after 2 days, significant differences were only evident for RUNX2 between DLP and FFF, while after 2 days, ALPL expression was not significantly different between the zirconia surfaces. After 1 day, no significant differences were observed for RUNX2. For ALPL, however, significantly higher expression was observed for cells cultured on MIL surfaces relative to that for the cells cultured on FFF and DLP surfaces.

Since we assumed there was stage-specific expression of the marker genes examined, we refrained from statistically comparing RUNX2 and ALPL expression at 7 days and 14 days. However, it is noteworthy that the kinetics of the declines in the expression of the early markers RUNX2 and ALPL are slightly different between surfaces; for example, cells on the FFF surfaces showed a faster decline in RUNX2 and ALPL expression than cells on the MIL surfaces, whereas cells on the DLP surfaces, regarding both markers, showed no further decline between 7 and 14 days. With osteocalcin (BGLAP) (Figure 7c), we also examined a late marker of osteoblast differentiation. Therefore, the observed peaks of expression after 7 and 14 days are within the expected range. Overall, osteocalcin expression was lowest after 7 and 14 days in the cells on the MIL surfaces. Significant differences were therefore seen after 7 days between DLP and MIL and after 14 days between MIL and DLP and FFF and between FFF and DLP. The highest osteocalcin induction was observed in the cells on the FFF surfaces; these were also the cells in which the peak of expression was observed at the latest examination time point (14 days). Overall, the observed temporal pattern of the expressions of the stage-specific markers indicates regular osteogenic differentiation of the cells; however, it also points to a more pronounced final differentiation on the FFF and DLP surfaces, while expression of the early differentiation markers appeared to be more supported by MIL surfaces, at least within the limited observation period.

## 4. Discussion

The results of this study suggest that additively manufactured zirconia, produced via Digital Light Processing or Fused Filament Fabrication, without any further surface modification offers cytocompatible surfaces that might be promising alternatives for the production of individualized dental implants.

Regarding DLP and MIL, our results are in line with the findings of our previous study, in which we compared both samples with equally polished surface structure roughness and for which we found comparable to slightly better results for DLP [33]. It should be considered, however, that the lowest proliferation rates were found for unpolished DLP surfaces both in our previous studies and in this study, indicating that polishing DLP surfaces enhances proliferation rates, at least for hfOB 1.19 cells. However, one should consider, however, that comparable results in terms of cell coverage and cell adhesion and even higher expression rates of the differentiation markers ALPL, RUNX2, and BGLAP were found for the DLP samples in both this study and our last one.

Cell coverage on a surface is determined by several interrelated factors, including the number of adherent cells, their proliferation rates, and the extent of individual cell spreading, which defines the area each cell covers. In this study, reduced cell adhesion after 24 h on the MIL and DLP surfaces likely contributed to the lower overall coverage observed, as fewer cells were available to populate the surfaces. In contrast, for the FFF surfaces, cell coverage was reduced despite relatively stable cell adhesion, suggesting that smaller individual cells and limited spreading are the primary factors. These findings underscore the influence of surface topography on cell morphology and behavior. However, since no statistically significant differences in cell coverage were detected between the surfaces at either time point, the observed trends do not provide a robust basis for distinguishing the effects of the different manufacturing techniques on cellular responses in terms of cell coverage.

It remains unclear whether lower proliferation rates justify the need for post-manufacturing polishing, which constitutes an additional post-printing process, limiting the clinical applicability of DLP. Therefore, further preclinical and clinical studies must be conducted in order to compare DLP samples with different surface structures.

In this study, we extended our investigations to FFF samples and found remarkable results indicating that this production technique might be highly promising for the production of dental implants. Compared to both the milled and DLP-printed samples, the FFF sample showed comparable results in terms of cell coverage and the expression of the initial differentiation markers RUNX2 and ALDL and even better results in terms of cell proliferation, cell adhesion after 24 h, and the expression of the terminal differentiation marker BGLAP. Regarding the expression of differentiation markers, we expected superior results for FFF in regard to the expression of the terminal expression marker BGLAP since an increase in surface roughness has been shown to promote cell differentiation [34,35,36,37]. These results are in line with previous work on FFF Polyetheretherketone (PEEK) samples [38] and might be attributable to the considerably higher surface roughness values found for FFF samples in comparison to the two other samples. One should keep in mind that differences in surface roughness values can be influenced by the measurement technique employed, which was white-light interferometry in this study. Measurements obtained via stylus profilometry typically yield slightly lower roughness values than white-light interferometry, as the contact-based method may partially smooth over fine surface valleys [39].

The differential expression of osteogenic markers observed in this study reflects the intricate interplay between surface topography, cell adhesion dynamics, and the subsequent stages of osteoblastic differentiation. The temporal expression profiles observed suggest that while the smoother MIL and polished DLP surfaces supported initial differentiation events, the rougher FFF surfaces promoted later stages of maturation. This progression likely stems from differences in cell–surface interactions that influence cytoskeletal organization and mechanotransductive signaling pathways, such as FAK and MAPK activation, which are known to regulate osteogenic gene expression [40,41].

In agreement with these molecular findings, the cell coverage data support the notion that surface roughness modulates not only adhesion but also cell spreading and intercellular connectivity. The FFF samples exhibited stable adhesion yet reduced coverage at early time points, potentially indicating limited initial spreading due to pronounced microtopographical features. Such conditions can restrict early proliferation but also enhance mechanosensitive signaling conducive to osteogenic differentiation. In contrast, the smoother MIL and DLP surfaces facilitated more uniform cell coverage but showed comparatively lower expression of late-stage markers, suggesting that flatter morphologies favor proliferation over maturation. Similar relationships between surface microstructure, adhesion dynamics, and osteogenic gene expression have been reported for titanium and zirconia substrates [37,42].

Taken together, our findings support the interpretation that the distinct surface architectures generated by different additive manufacturing techniques differentially direct osteoblast behavior. While the smoother, polished DLP and MIL surfaces promote early-stage adhesion and proliferation, the inherently rougher FFF surfaces appear to drive more advanced differentiation toward the osteoblastic phenotype.

The high surface roughness observed is caused by the defined strands coming out of the nozzle during printing. To achieve the highest densities and mechanical strength, the samples are usually overfilled to avoid the creation of voids between the printed lines. Through overfilling, more material than necessary for printing is transported, and residual feedstock is pushed to the surface. Although the majority are wiped by the nozzle, some bumps induced by overfilling remain, leading to high surface roughness [43,44,45]. By varying the printing parameters, like nozzle diameter and layer height, one can modify the characteristic surface structure, which is independent of the material used. Wang et al. increased roughness by reducing the distance between the printing bed and nozzle without reducing material flow [46]. This led to an excess of material that must displace to the surface on the top. Decreasing layer height and improving the welding of the layers can decrease roughness [47,48,49]. Regarding the influence of surface modification on cytocompatibility, Shilov et al. studied different 3D-printed polymers with diverse printing parameters [50]. In their study, the cytocompatibility results varied considerably depending on the printing parameters for two of the three polymers tested. Interestingly, surfaces produced via FFF printing showed tended to be associated with improved cell responses, a result that may be due to the distinct surface characteristics inherently generated by the FFF process. Thus, compared to mechanically treated (milled) or DLP-printed surfaces, the intrinsic surface texture of FFF-printed materials could offer an advantage in terms of cytocompatibility.

Although FFF demonstrates promising cytocompatibility, it is important to recognize that appropriate mechanical properties are equally critical for dental implants. PEEK serves as a pertinent example in this context. While it shows favorable cytocompatibility [50], its mechanical strength is compromised by insufficient interlayer adhesion, a limitation inherent to the FFF process. In contrast, ceramics like zirconia undergo a sintering step, which promotes interlayer fusion and reinforces the structurally weak regions perpendicular to the direction of the applied pressure. Nonetheless, even in ceramics, process-related voids and surfaces irregularities can impair mechanical performance [51]. For instance, Zhang et al. evaluated additively manufactured implants with highly porous surfaces in accordance with ISO 14801 [52] and reported a lower fracture load compared to conventionally manufactured implants that did not undergo surface modification. Interestingly, they observed that poorly ground samples (ground using grinding paper #220), which exhibited greater surface roughness, did not show significantly reduced flexural strength compared to polished samples (ground using grinding paper #1200) with smoother surfaces [23]. Nevertheless, the measured bending moments exceeded thresholds considered clinically safe [53]. The literature on this topic remains highly heterogenous, particularly regarding implant surface treatment, aging protocols, and mechanical testing methodologies [54]. Some studies have reported that surface modifications have beneficial effects on the fracture resistance of ceramic implants after aging, while others indicate detrimental outcomes. Given that mechanical strength must comply with established standards for clinical use, future studies should include comprehensive mechanical testing of FFF-printed and sintered samples to assess their suitability as dental implants.

When interpreting the results of this study, several limitations must be considered. First, since this is an in vitro study, direct conclusions regarding the clinical performance of these materials are limited, and therefore further studies, particularly on FFF-ZrO2 with different surface modalities, are required. Moreover, for our investigation, we used hfOB 1.19 cells since they are established tools for the investigation of the osteogenic potential of various materials [55,56,57]. The human fetal osteoblast cell line hFOB 1.19 was selected for this study due to its high proliferative capacity, long lifespan, and well-characterized osteoblastic phenotype, including robust expression of alkaline phosphatase and osteocalcin and matrix mineralization in response to differentiation cues [32]. This standardized model circumvents issues regarding donor variability and replicative senescence inherent to primary adult osteoblasts or bone-marrow-derived MSCs, enabling reproducible analysis of fundamental cellular mechanisms. Additionally, hFOB 1.19 cells are widely used to evaluate the cytocompatibility and osteoinductive potential of biomaterials, demonstrating appropriate adhesion, proliferation, and differentiation on diverse matrices [57,58]. However, using other cell lines might affect the results [36].

Furthermore, we acknowledge that fetal osteoblasts may respond differently relative to adult skeletal progenitor cells, which possess distinct developmental and functional properties relevant to bone regeneration. Nevertheless, we believe that the use of hFOB 1.19 cells is justified for mechanistic in vitro studies, as they provide insight into osteoblast-specific regulatory processes, while the responses of primary adult osteoprogenitor cells clearly need to be investigated further for translational validation.

We also want to highlight the critical issue of balancing the osseointegration benefits of increased surface roughness with the microbiological risks associated with excessive roughness at the implant neck, particularly in the context of FFF (Fused Filament Fabrication) implant design. Such roughness at the transmucosal or neck region of the implant, which is exposed to the oral environment, can facilitate bacterial adhesion and biofilm formation [59]. This inherent trade-off between osteogenic and microbiological requirements is not a challenge that is unique to FFF surfaces, as it is also encountered with gold-standard blasted and acid-etched titanium implants [60]. Site-specific modification strategies, such as selective smoothing of critical transmucosal areas, may offer a viable compromise, preserving roughness in regions essential for osseointegration while mitigating microbiological risk. This approach may be particularly advantageous for complex implant geometries as intended for our FFF implants, allowing for local adjustments of surface topography in order to cope with microbiological requirements without globally reducing osteogenic potential.

## 5. Conclusions

Additive manufacturing of zirconia using Fused Filament Fabrication appears to be a promising approach for dental implant fabrication, particularly due to the inherent surface roughness, which may favorably influence support cellular responses. In the absence of additional surface treatments, both FFF- and stereolithography-printed zirconia demonstrated overall cytocompatibility. FFF showed comparable results in terms of cell coverage and early-osteogenic-marker expression (RUNX2 and ALPL), with indications of enhanced cell proliferation, adhesion, and late-stage differentiation (BGLAP). These findings suggest that FFF may hold potential for the development of patient-specific, highly biocompatible implants. However, further investigations—particularly into mechanical properties and long-term clinical performance—are warranted to confirm this technique’s suitability for clinical applications.

## Figures and Tables

**Table 1:** RT^2^ qPCRPrimer assays for quantitative PCR.

Target	Assay ID (Gen Globe ID)
human GAPDH	PPH00150F
human RUNX2	PPH01897C
human ALPL	PPH58134F
human BGLAP	PPH01898A

**Table 2:** Surface roughness values for the different groups (±0.025 µm).

Samples	Mil	DLP	FFF
Sa [µm]	0.3	0.2	5.5
Sq [µm]	0.4	0.3	10.8
Sp [µm]	3.4	1.8	34.2
Sz [µm]	6.3	8.1	132.3

## Data Availability

The complete raw data supporting the findings of this study are available from the corresponding author upon reasonable request.

## Abbreviations

The following abbreviations are used in this manuscript:

ZrO_2_	Zirconia
Ti	Titan
CNC	Computer numerical control
AM	Additive manufacturing
DLP	Digital light processing
FFF	Fused filament production
RUNX2	Runt-related transcription factor 2
ALPL	Alkaline phosphatase
BGLAP	Osteocalcin (“Bone γ-carboxylglutamic acid-containing protein”)
Mil	Milled
SA	Stearic acid
LA	Lauric acid
PVB	Polyvinyl butyral
PEG	Polyethylene glycol
hFOB	Human fetal osteoblast
DMEM	Dulbecco’s Modified Eagle Medium
OD	Optical density
PBS	Phosphate-buffered saline
HMDS	Hexamethyldisilazane
SEM	Scanning electron microscopy
BSED	Backscattered electron detector
ROI	Region of interest
qRT-PCR	Quantitative reverse transcription polymerase chain reaction
cDNA	Complementary DNA
M-MuLV RT	Moloney murine leukemia virus reverse transcriptase
poly-dT	Polydeoxythymidine
ΔΔCT	Delta-delta CT
RMS	Root mean square
PEEK	Polyetheretherketone
